# High temporal resolution data reveal low bat and insect activity over managed meadows in central Europe

**DOI:** 10.1038/s41598-024-57915-0

**Published:** 2024-03-29

**Authors:** Melina T. Dietzer, Lara Keicher, Jenna E. Kohles, Edward R. Hurme, Ireneusz Ruczyński, Tomasz Borowik, Marcin Zegarek, Mateusz Choiński, Dina K. N. Dechmann

**Affiliations:** 1 Department of Biology, Universität Konstanz, Konstanz, Germany; 2 Department of Migration, Max Planck Institute of Animal Behavior, Radolfzell, Germany; 3 Department of Wildlife Ecology and Management, Universität Freiburg, Tennenbacherstraße 4, 79106 Freiburg, Germany; 4 Mammal Research Institute PAS, Białowieża, Poland; 5 Cluster for the Advanced Study of Collective Behaviour, Universität Konstanz, Constance, Germany; 6 Faculty of Computer Science, Bialystok University of Technology, Białystok, Poland

**Keywords:** Biodiversity, Behavioural ecology, Conservation biology, Grassland ecology, Biodiversity

## Abstract

Increasing agriculture and pesticide use have led to declines in insect populations and biodiversity worldwide. In addition to insect diversity, it is also important to consider insect abundance, due to the importance of insects as food for species at higher trophic levels such as bats. We monitored spatiotemporal variation in abundance of nocturnal flying insects over meadows, a common open landscape structure in central Europe, and correlated it with bat feeding activity. Our most important result was that insect abundance was almost always extremely low. This was true regardless of management intensity of the different meadows monitored. We also found no correlation of insect abundance or the presence of insect swarms with bat feeding activity. This suggests that insect abundance over meadows was too low and insect swarms too rare for bats to risk expending energy to search for them. Meadows appeared to be poor habitat for nocturnal flying insects, and of low value as a foraging habitat for bats. Our study highlights the importance of long-term monitoring of insect abundance, especially at high temporal scales to identify and protect foraging habitats. This will become increasingly important given the rapid decline of insects.

## Introduction

A worldwide decline of insect abundance and species diversity has been observed in past years and is most likely linked to extensive use of pesticides and monocultures in agriculture^1–4^. Empirical quantifications of this decline are largely lacking^5^. Currently, one of the main limitations is that most insect monitoring is conducted across a narrow range of environments, and often in areas that are already considered ecologically valuable. Many studies are carried out only on the short-term, and focus on insect diversity and or pollinators^6,7^. Insects are most often captured or monitored in a cumulative way via transect walks with sweep netting or pitfall, pan, malaise or suction traps, and collected for later identification^8–10^. While these methods are important to understand ongoing changes, insect biomass is often equally important as an ecosystem trait, especially when considering their importance as food for species at higher trophic levels. In addition, most methods require time consuming identification of captured insects, limiting the temporal and spatial scale at which studies can be carried out. Looking forward, it is necessary to expand, and in many places still begin, long-term monitoring of insects across a wide range of habitats using standardized approaches, incorporating spatio-temporal patterns of insect abundance.

Such long-term insect monitoring is urgent as insect declines have manifold consequences^11^. Insects are essential for ecosystems, as pollinators or as food sources for many animal species^12^ among many other functions, providing large economic value^13,14^. Furthermore, declines of insect abundance are not the only problem. Many regions are experiencing ecological homogenization of insect communities, where a diversity of species is replaced by greater numbers of the most common species, often those with higher tolerance for environmental change, and many of which are agricultural pests^4,5^. As the proportion of insect pest species increases, the need for biological control via insectivorous predators such as birds or bats also increases, as chemical control of agricultural pests exacerbates the problem further. Studies looking at cascading effects between trophic levels during declines in diversity often focus on invertebrate species communities, but recent work suggests that high trophic levels, especially of vertebrates, are crucial for maintaining system stability and carbon cycles^15^. However, insectivorous vertebrates are in decline, a phenomenon best studied in birds^16,17^. Thus, it is crucial to monitor insectivorous predators as well as their insect prey to generate the knowledge basis for management plans that regulate and maintain both.

The majority of the more than 1400 global bat species are insectivorous^18^. They are present on all continents except Antarctica, and often adapted to very specific habitats and prey^19^. Destruction or alteration of their habitats and prey can reduce bat populations^20,21^ and have cascading effects as large and stable bat populations are crucial to maintain the ecosystem services they provide, for example as predators of seasonal surges of agricultural or forest pests^22,23^. This is particularly true for many European aerial-hawking bat species which favor clutter-free environments when foraging due to their long and narrow wing shape. Nearly all of these species opportunistically exploit ephemeral swarming insects, a clumped and energy-rich but short-lived resource^24^. In central Europe, most open, forest-free habitats available to bats occur over land used for agriculture, grazing or haymaking. Yet the intensifying use of these landscapes has been correlated with declines in both invertebrate and vertebrate species across taxa, including bats^25–27^.

Meadows are one common open habitat in central Europe, and they are managed with varying intensity. They are often mowed without taking into account insect life history cycles, usually fertilized, and plant diversity is low. Consequently, such meadows may vary greatly in insect species diversity. However, it remains unknown if overall insect abundance is also low in these meadows or if they may still serve as a good food source for some bat species. Many studies on insect abundance and species diversity focus on wetlands, the more valuable non-agricultural open landscapes, and monitoring of insects on meadows as a potential food source for insectivorous bats is lacking^28,29^. In particular, there is little information on how the abundance of flying insects varies in time and space, information that is crucial for understanding the value of these insects as a food resource of a given predator taxon, such as aerial-hawking insectivorous bats, which forage only at night and often depend on swarming insects^25,30^.

In this study we used cameras to monitor abundance of nocturnal flying insects over meadows in Southern Germany in summer. Meadows varied in the manner and intensity with which they were managed, and we investigated the influence of grass height as well as weather, time of night and day of year, on insect abundance. Simultaneously, we monitored foraging activity of bats using ultrasonic acoustic recordings. We hypothesized that (1) insect abundance would seasonally peak in July and around sunset each night, be positively correlated with temperature and grass height, but negatively correlated with wind speed. However, we also hypothesized that (2) overall insect abundance and especially the occurrence of insect swarms would be low over meadows, and negatively correlated with increasing intensity of management. Finally, we expected that (3) we would primarily record bat activity from species that are adapted to foraging in open spaces (in contrast to edge or clutter specialists), but if insect swarms were as rare as predicted, that bat feeding activity would also be low and not correlate with presence of insect swarms. The results of our study will contribute to our understanding of insect availability in human-altered landscapes, and link this to activity of species at higher trophic levels that depend on insect prey.

## Results

### Insect abundance

Nocturnal flying insect abundance was measured from ten different meadows across 33 different nights (some meadows were monitored simultaneously on the same night). In total we used 95 meadow-nights for analyses. The meadow “Mill” exhibited the highest number of insects detected on a single photo, with a count of 105 insects. On the other hand, the meadow “Uni” had the highest mean number of insects per photo, averaging 1.79 insects per photo. In contrast, the meadow “Wollmatingen” had the lowest mean number of insects per photo, with only 0.3 insects observed on average (see supplementary Table S1 for details). All meadows were managed differently, and ranged from sheep briefly grazing twice per year, to intensive fertilization with several mowing events per year. We analyzed 42,324 photos and 31.3% (n = 13,426) of the photos contained insects. Generally, insect numbers on all meadow-nights were low and swarms were so rare that their occurrence could not be modeled. The number of insects in a photo ranged from 0 to 105. Of the photos that were not empty of insects, almost half (49.9%) contained only one insect. Insect swarms, defined as ten or more insects^31^, occurred in only 2% (n = 845) of photos.

### Model results

**Figure 1:**
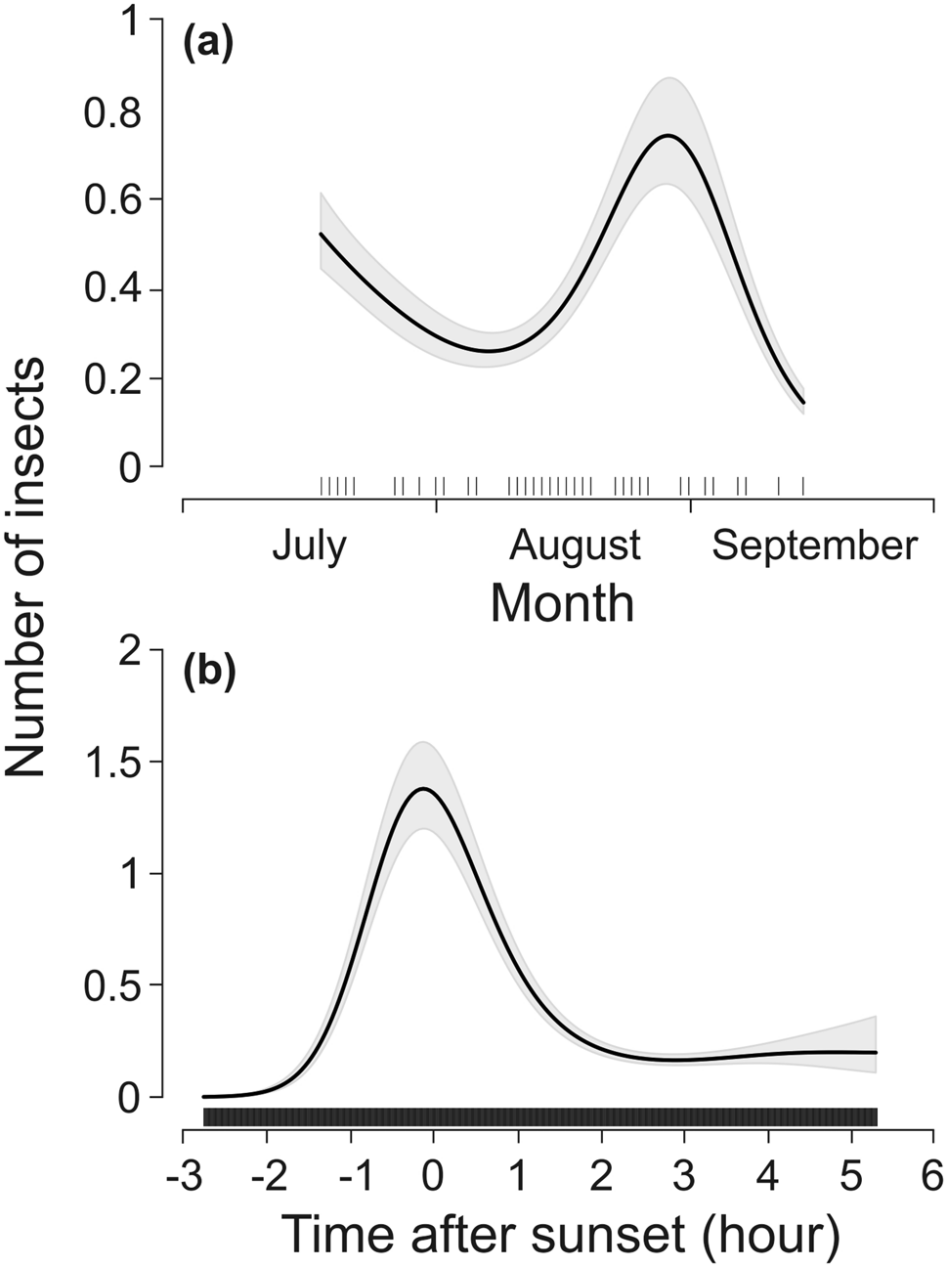
Insect numbers (recorded per minute) across (**a**) months and (**b**) hour after sunset. Shadowed gray areas represent 95% confidence intervals. The distributions of the explanatory variable values are marked with black vertical lines on the x-axis.

Model results are summarized in Table 1. When insect abundance was examined, a small but significant difference in the number of insects was found between the center and the edges of the meadow, i.e., there were more insects on the platforms in the center of the meadow than on those at the edges (Supplementary Fig. S1b). No difference was found between years (Supplementary Fig. S1a). As expected, there was a seasonal insect peak, but it occurred in late August, later than expected (Fig. 1a). Insect numbers were highest at sunset (Fig. 1b).

**Table 1:** Association between number of insects in photos and wind speed, temperature, grass height, time of the year, and time after sunset in Southern Germany in 2020 and 2021.

Variable	Estimate ± *SE* or *edf*	*z*-value or *Χ*^*2*^	*P*
Parametric terms			
Intercept	− 0.57 ± 0.20	− 2.90	0.004
Year: 2021 (2020)	0.06 ± 0.04	1.30	0.19
Location on meadow: middle (edge)	0.05 ± 0.02	2.35	0.02
Smooth terms			
Wind	3.91	448	< 0.001
Temperature	3.81	1488	< 0.001
Grass height	3.98	495	< 0.001
Time of the year	3.96	695	< 0.001
Time after sunset	3.99	4410	< 0.001
Meadow ID^a^	8.90	732	< 0.001

Results of GAM1. Reference levels are presented in parenthesis.

^a^Random factor.

**Figure 2:**
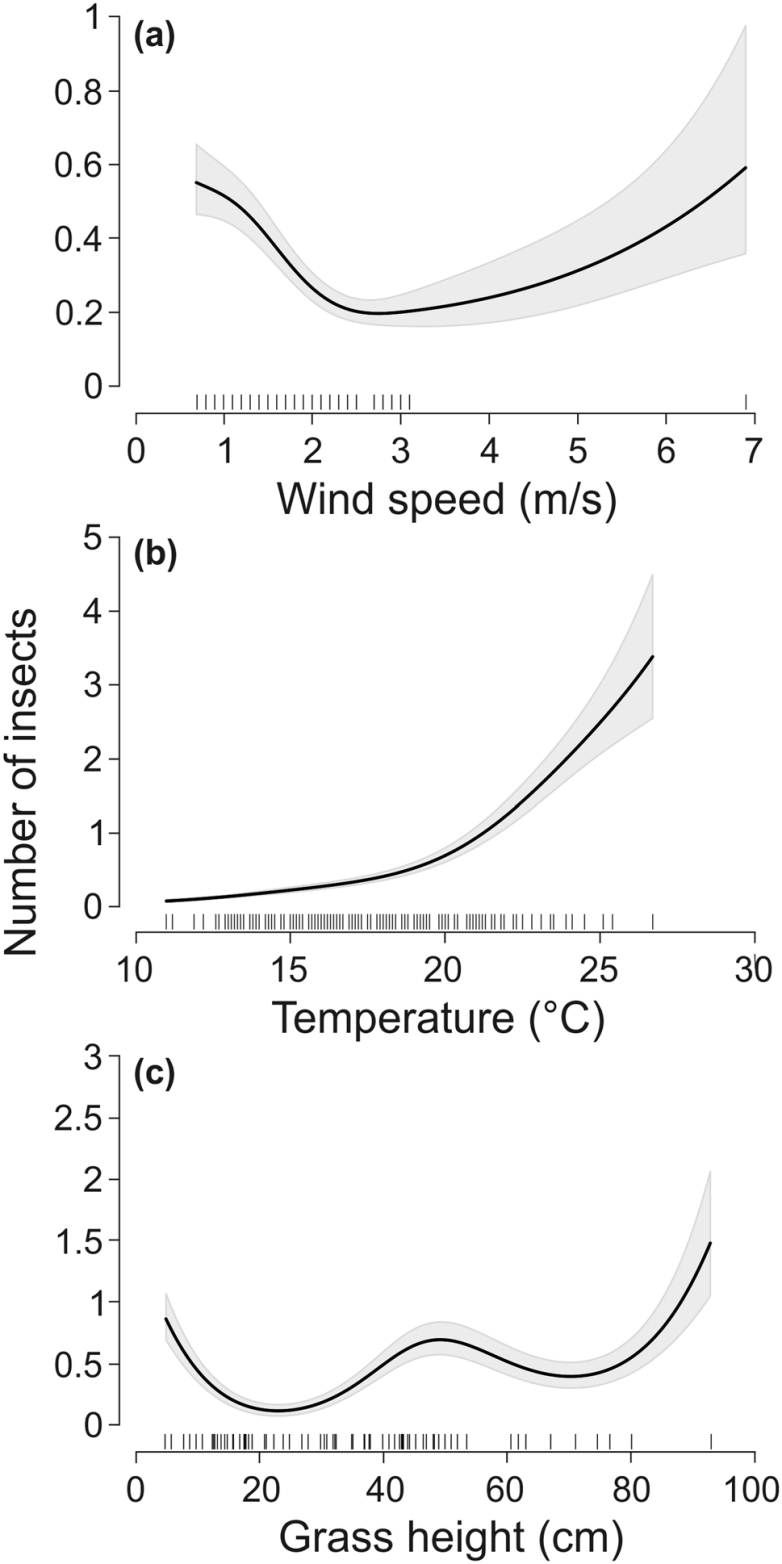
Effect of (**a**) wind speed, (**b**) temperature and (**c**) grass height on insect numbers. The shadowed gray areas represent 95% confidence intervals. The distributions of the explanatory variable values are marked with black vertical lines on the x-axis.

Insect numbers decreased as wind speeds increased from 1 to 3 m/s and increased again up to wind speeds of 7 m/s (Fig. 2a). Insect numbers increased with higher temperatures, especially at temperatures higher than 20 °C (Fig. 2b). Insect numbers were slightly higher around a medium grass height of approximately 40 cm (Fig. 2c), however, our sample size at higher grass heights was low.

### Bat feeding activity

Bat feeding activity was measured in 21,793 1-min long recordings from eight different meadows from 30 different nights (corresponding to 88 meadow-nights) over the two sampling years. Only 0.8% (n = 185) of the recordings contained one or more feeding buzzes. The mean number of bat feeding buzzes recorded per night ranged from 1.4 to 15.5 (Supplementary Table S2). Buzzes were produced by *Pipistrellus pipistrellus* (44.8%), *Nyctalus noctula* (33.5%), *Pipistrellus pygmaeus* (17.5%) and *Pipistrellus nathusii/P. kuhlii* (4.1%). *Pipistrellus pipistrellus* produced the most feeding buzzes overall, but *N. noctula* emitted the most feeding buzzes in the meadow with the highest average number of feeding buzzes (“Hockgraben”). There was no correlation between insect swarms and the presence of feeding buzzes (Table 2). Data were not sufficient to test if single species responded to the occurrence of insect swarms.

**Table 2:** Relationship between insect swarms (more than 10 insects in at least one photo out of 5, taken within 5 min) and the probability of bat buzzing.

Variable	Estimate ± *SE* or *edf*		*z*-value or *Χ*^*2*^	*P*
Parametric terms				
Intercept	− 3.71 ± 0.26		− 14.2	< 0.001
Swarming: presence (absence)	− 1.01 ± 1.01		− 1.00	0.32
Smooth terms				
Meadow ID^a^	4.79		116	< 0.001

Results of GAM2. Reference levels are presented in parenthesis. ^a^Random factor. Data from eight meadows could be used in this analysis as two of the ten meadows did not have valid acoustic data.

## Discussion

Insect abundance over meadows in Southern Germany was nearly always low, regardless of how intensively a meadow was managed. Mean insect abundance per minute ranged from zero to five insects, and even small insect swarms were extremely rare. The fact that feeding buzzes did not correlate with insect swarms suggests that insect numbers on the meadows sampled in this study were so low that they did not provide a sufficient foraging habitat for European insectivorous bats, regardless of management intensity, time of year, night time, grass height, or weather.

There is a considerable body of research investigating insect abundance in different habitats and ecosystems. Some of these studies have shown that semi-natural grasslands exhibit high species diversity and abundance of pollinating insects^32^, while others have demonstrated that intensively managed grasslands have lower species richness and abundance of pollinating insects^33^. In our study of managed meadows, the number of insects in photos was almost always low.

Insect biodiversity can be improved by the way meadows are managed^34^. However, natural and semi-natural meadows and low-intensity farmland which provide a much better habitat than managed meadows are rare in spatial distribution^35^. Our findings of low insect abundance emphasize the importance of evaluating the influence of various management practices on insect populations in managed meadows. Although the type and intensity of management practices, as well as the specific mowing techniques employed, can have profound effects on insect diversity and abundance^36^. The presence of flowering plants^37^, surrounding landscape^38^, or leaving grasslands semi-natural^39^, may play a role in insect biodiversity conservation. Continuous monitoring of managed meadows and associated mowing practices is essential to follow the trends in insect diversity and abundance in managed meadows. Only by systematically assessing the responses of insect populations to different management approaches, one can gain valuable insights into what actions are needed to increase the attractiveness of these meadows to insects and their predators.

We found that meadows with minimal human impact, such as the wet meadow “Mill” exhibited the highest insect abundance on the photos. Compared to the meadow “Mill”, the three meadows “Tannenhof”, “Hockgraben” and “St. Katharina” show lower insect numbers. This may be due to their intensive management, including frequent mowing and fertilization. The meadow “Hegne” displayed the lowest insect abundance. Although it is situated in a favorable environment near a lake, it undergoes intensive management practices and the use of fertilizers. Additionally, the proximity of a nearby road may have additional implications for the insect population. Interestingly, the “Institute” meadow, despite being grazed solely by sheep and considered a natural meadow, showed a remarkably low number of insects. This could be attributed to the strong sunlight exposure and aridity of the location. Our findings emphasize the importance of environmentally conscious meadow management to promote insect diversity. A previous study in Poland^31^ using the same method as we did, found lowest insect abundances in forest clearings, which are probably most comparable to our meadows, and higher abundances in wetlands which are most comparable to our wet meadow. Insect swarms were similarly rare in Poland as in Germany, but were much larger when they occurred, especially over lakes. More monitoring of nocturnal insects across a larger range of habitat types and at a broader geographical scale is needed to confirm these patterns.

We found a significant seasonal increase in insect abundance from early to late August across all meadows (Fig. 1a). The occurrence of insect swarms increased rapidly from the beginning to the middle of August and then decreased throughout late August. Interestingly, in eastern Poland, Ruczyński et al.^31^ showed a decrease in insect swarming probability after a peak in early July. After winter hibernation, the females of some European bat species migrate in early summer from Central Europe to northeastern Europe to complete pregnancy and raise their young^40^. They then return to their hibernation areas in August.

Empirical research has consistently found that bats match their activity patterns to those of insects, with a peak of both occurring around and just after sunset including over meadows. We also found that insect abundance including swarms peaked around sunset and for up to one hour after sunset, then subsequently decreased (Fig. 1b). This is consistent with the results of previous studies^31,41,42^ and seems to be the prevailing pattern regardless of total insect abundance or habitat type. Insects seem to emerge at sunset and/or aggregate in mating swarms during this time of day, and thus partly avoid predation by birds (which are only active during daylight) and bats (which emerge around or after sunset)^43^. However, in our studies the numbers of insects across most meadows were so low that a matching peak in bat activity was not observed.

Our model shows a strong positive effect of increasing temperature on insect abundance (Fig. 2b). Likewise, the sharp decline in insect abundance at our study sites in late August is most likely caused by a decrease in ambient temperatures^31,43,44^. Insect abundance decreased up to wind speeds of 3 m/s. Above this speed insect abundance increased, but wind speeds over 3 m/s rarely occurred during our sampling period (Fig. 2a). In Sweden, the abundance of flying insects occurring above wind farms were equally abundant at mild breezes (3 m/s) up to moderate gales (7 m/s), but larger insects were more abundant during lower wind speeds^45^. In our study, wind speed was not directly measured at each meadow, but at the closest weather station, and variation in wind speed was in general low, so interpretations are limited.

Insect abundance was also influenced by grass height (Fig. 2c). Fewer insects occurred at low grass heights of > 10 cm than in meadows with grass heights up to 40 cm. According to our model, insect abundance also decreased at grass heights above 40 cm, but our meadows were usually mowed before they reached these or larger heights and we cannot substantiate this result. Although this has rarely been quantified, birds often arrive quickly on mowed meadows, presumably as both invertebrate and vertebrate prey are disturbed by the mowing processes and become easy targets for predators^46–48^. When we tested for the effect of mowing (data not shown) we found no effect. This is likely due to the effect of insects being “whirled up” in large quantities due to mowing lasting only a short time. As meadows were mowed during the day, and the insects likely left the meadows or settled again by the time our monitoring began at sunset we did not directly observe it. Insectivorous bats have been reported to hunt over grain fields in Poland that are harvested at night^47^. Such night harvesting events, which produce light, noise, and a strong odor might be easily detected from a long distance and may provide an important resource for bats, a hypothesis which warrants further study.

The bat species with greatest feeding activity over meadows were several species of *Pipistrellus* and *Nyctalus noctula* (Supplementary Table S2). However, high bat feeding activity was not always associated with high insect abundance. The meadow with the highest average number of feeding buzzes per night (15.5 buzzes), had feeding buzzes produced mostly by *P. pipistrellus* and *P. pygmaeus*, and had lower than average insect abundance. In contrast, the meadow with the second highest average number of feeding buzzes per night had the greatest average insect abundance, and buzzes were produced by all bat species including the open space forager, *N. noctula*. This second meadow was larger and occurred within a larger area of open space, whereas the first had forest in closer proximity. Some *Pipistrellus* species are more specialized in foraging along forest edges or in forest gaps, and less strongly specialized on swarming insects^49^ unlike *N. noctula* which forages exclusively in open space without clutter^49,50^. However, *P. pipistrellus* is a flexible species and can also forage over open areas close to forest structures. It is also possible that we recorded some *Pipistrellus* buzzes while they were foraging in adjacent areas rather than directly over our open meadows. This might explain why insect abundance could be low while buzzes were high. Although meadows may still be important for *Pipistrellus* species particularly because they create important edge habitats when they occur alongside forests, these results suggest that monitoring bat activity via presence of echolocation calls without simultaneously monitoring insect abundance, as is often done, may bias our perceptions of species-specific habitat use and ultimately our understanding of bat foraging ecology.

There is a large and growing body of literature on insect declines^3,51^ including studies on downstream effects on other invertebrates. Yet studies directly linking the effects of insect abundance to vertebrate feeding activity and ultimately foraging success are lacking, especially for nocturnal species such as bats. Bats are frequently monitored via acoustic surveys of their echolocation calls to indicate their presence or absence in an area and this provides invaluable data on bat species’ use of different habitat types, but says very little about whether a habitat type is particularly valuable for bats in terms of providing food resources. While feeding buzzes are only evidence of a capture attempt, not necessarily a successful capture, they are only produced when bats detect prey and thus a good indicator of what the bat is perceiving. Reliable automated detection of feeding buzzes is in progress, but not yet easily possible, unlike automated detection of species-specific echolocation calls. Analyzing acoustic recordings not only for species-specific echolocation calls, but also for feeding buzzes which indicate bat feeding activity, can start to fill this gap in assessing links between low insect abundance and predators of insects. Our study suggests that low insect abundance renders meadows a habitat of poor quality for bats, even though along with agricultural monocultures they are one of the few remaining open habitat types in many landscapes.

The management practices and the surrounding environment of meadows can have significant impacts on insect populations, which in turn can affect ecosystem functioning and services. In this study, we analyzed eight meadows with varying management practices and environments, including “Mill”, “Hockgraben”, “Tannenhof”, “Scheune”, “Voliere”, “St. Katharina”, “Uni”, and “Institut”. Management of meadows ranged from rare mowing or grazing by sheep without the use of pesticides to intensive mowing or harvesting with the use of pesticides. Despite the varying management practices, environments, and use of pesticides, our study found little difference in the occurrence of insects among the meadows.

In summary, our study shows that open meadows in Southern Germany produce and/or support very low numbers of insects across a range of management regimes. Nevertheless, studies have shown that the level of management intensity of meadows can significantly affect insect abundance. Typically, more intensive management practices are associated with lower insect diversity and abundance^52^.

Heterogeneity of the surrounding landscape can also have significant effects on insect populations^53^. The presence of adjacent forests, wetlands and good vegetation quality can offer essential resources for insects, which may increase their abundance in meadows^54–56^. We also quantify the cascading effect of very low insect abundance in managed meadows in southern Germany across a range of management regimes on the foraging success of an entire group of insect-dependent predators, bats.

Once insect availability and especially the frequency and size of swarms drops below a certain threshold, it seems that bats are unable to exploit them, as they must find and feed on enough insects to offset high costs of flight. With ongoing insect declines it is important to monitor insects across a range of habitats and at good spatiotemporal resolution to assess their value for the predators that depend on them.

## Methods

### Study site and sampling regime

We monitored nocturnal flying insect abundance and bat activity on open meadows. The selection of the meadows was based on aerial images from Google Maps. We tried to select all meadows that at least one side of the meadow borders on a forest edge or a larger group of trees. The surroundings of the meadows vary, so some are located near large bodies of water, others near bigger forest patches and some near more urbanized areas.

The average distance between meadows was 1097 m, with a standard deviation of ± 1337.38 m. The maximum distance observed between meadows was 3870 m, and the smallest distance recorded was 227 m. Data were collected from August to October 2020 and from July to September 2021 on ten meadows (1100–33,500 m^2^, see supplementary Table S1 for details) that varied in management intensity. Management ranged from sheep briefly grazing twice per year, to intensive fertilization with several mowing events per year. Meadow “Mill” was managed through either annual mowing or grazing by sheep without the use of pesticides. Similarly, “Institut” was grazed by sheep and pesticide-free, located on the edge of the forest and road, and in close proximity to a residential area. “Voliere” was pesticide-free and mowed twice a year, surrounded by trees, and situated on the edge of the forest. The partially used meadow “St. Katharina” was intensively used but without the use of pesticides, situated in a clearing within the forest. Similarly, the meadow at the University, “Uni” and “Hockgraben” were intensively used but free of pesticides, located on the edge of the forest and close to the university campus. “Scheune” and “Tannenhof” were intensively used fields that employed the use of pesticides, with houses and a road located in the surrounding area and the forest edge a few hundred meters away. We did not assess plant diversity directly, but used management strategy as a proxy for how diverse we expected meadows to be.

We monitored meadows between four and eight nights each in each year. All meadows were also mowed during the sampling period and we used this to incorporate the effect of grass height on insect abundance (mowing as a direct effect tested, but not included here as it happened usually too early in the day to affect bat activity). On each sampling event, the number of flying insects and bat activity were continuously recorded in the 15 min before sunset to three hours after sunset. To measure insect abundance, we used digital cameras (Ricoh WG-5 GPS)^31^. We aimed the cameras towards the night sky and took a photo every one minute using flash. Flying insects were visible on the resulting pictures as white shapes on a black background. Each camera was placed on a two-meter-high platform and detects, small insects (wing length ≤ 4 mm) in volume of 18 m^3^ and a height of four meters and very large insects, such as Heterocera (wing length ≤ 15 mm) in a volume of 282 m^3^ and a height of 10 m^31^. Each camera took 195 photos per sampling event. We used AudioMoths (Open Acoustic Devices) to make simultaneous acoustic ultrasound recordings and monitor bat feeding activity. We recorded in full-spectrum uncompressed WAV format, saving recordings in 1-min wav files. We paired each AudioMoth with one camera and placed them on a two-meter-high platform to avoid artifacts caused by vegetation. AudioMoths were protected in a thin plastic zip bag, and placed on platforms with the battery side facing down and the microphone facing up. As AudioMoth devices are known to vary quite strongly, and detection ranges strongly depend on local conditions, no generally applicable literature on this is available. A direct assessment of detection ranges of the various species of bats in our study area would have been beyond the scope of this study. Species that echolocate at different frequencies are expected to have different detection probabilities^57^. As we were mostly interested in relative differences in time and space and the same devices were deployed randomly on all meadows, we believe our approach is sufficient.

One platform was placed at the edge of each meadow and one in the middle. The distance between the platforms was 50 m. This could not be maintained in one meadow (“Voliere”), where the distance between platforms was 23.1 m. Each meadow was monitored at least every two weeks, but the order was randomized to control for variation in weather and time. Up to three meadows were monitored in each sampling night and we did not collect data on nights with rain, as rain can strongly affect activity of bats^58^.

We measured grass height and other environmental conditions (ambient temperature, wind speed and moon phase) for each sampling night of each meadow. We estimated grass height by averaging 30 measurements of vegetation height along a diagonal transect across each meadow. Measurements were spaced roughly 1 m apart. Ambient temperature (at 2 m above the ground) and wind speed were obtained from "Deutscher Wetterdienst” (opendata.dwd.de, station Konstanz) with an hourly average.

### Data extraction

We only included sampling nights where at least 100 photos had been taken. We extracted insect counts per photo using a machine-learning algorithm (Choiński et al. in prep.) to quantify fine-scale temporal insect distribution in time and space. The insect counting method was developed and tested based on photos collected in Białowieża Forest, Poland^40^. A subset of photos (n = 2090) was counted manually to verify the accuracy of the automated counting software. Precision of the algorithm used was 0.819, with a recall rate of 0.826 and F1 Score of 0.822. Algorithm precision was optimized by comparing manual visual counting and automatic counting. To determine whether insects are available in quantities large enough to be exploited by bats we defined photos containing more than ten insects as representing insect swarms^31^.

We took advantage of a well-known property of bat echolocation and behavior to quantify feeding activity: bats produce a terminal feeding buzz vocalization for every insect that they localize and capture or attempt to capture from the air. Thus, we counted feeding buzzes in our recordings to measure bat feeding activity. To do so we converted all AudioMoth recordings into spectrograms (frequency = 20–80 kHz, window length = 1024) of 5-s duration and visually counted bat feeding buzzes in the images. On two meadows ("Hegne" and "Wollmatingen") electronic interference made acoustic analysis impossible, so they were excluded from this analysis. Bat species (*P. pipistrellus*, *N. noctula*, *P. pygmaeus* or *P. nathusii/ P. kuhlii*) were manually identified based on their call characteristics^59^ and assigned to feeding buzzes by selecting calls that occurred immediately before buzzing. As the call repertoire of *P. kuhlii* and *P. nathusii* overlaps almost completely and both species occur year-round in suitable habitats in Southern Germany, we treated these two species together. We then calculated nightly means of the number of buzzes for each meadow as well to see how many buzzes were produced by each recorded species of bat.

We found that platform position (middle, edge) had an influence on insect abundance but not on bat feeding activity, likely due to the fact that the detection range of the AudioMoths exceeded the distance between the two platforms. We thus calculated bat feeding activity by taking buzz counts of one randomly chosen platform for each meadow on each night. In rare cases, some devices (cameras or AudioMoths) failed to function during a sampling session. In these cases, we measured insect abundance and bat activity from the remaining functioning device without randomly selecting the data from one of two devices.

### Statistical analyses

To test the effect of temporal and environmental variables on insect abundance (number of insects recorded per minute), we applied a negative binomial generalized additive model (GAM1; ‘mgcv’ package)^60^. There was low multicollinearity among explanatory factors (*R* <|0.3|) thus all variables were included in GAM1. The model included the following explanatory variables: wind speed, temperature, grass height, month, time since sunset, year, location on the meadow (edge vs middle). We added meadow ID as a random effect (as a penalized regression term). We used 'year' as an explanatory variable to correct for seasonality, rather than looking at the seasonal effect. Unfortunately, we could not add this variable to the random effect part of the model because it only had two levels, whereas the recommendation for a random effect is to apply it to variables with at least 5–6 levels. We set the same smoothing for each continuous variable and we retained smoothing intensity (curvilinearity) at a relatively low level (k-index = 5) to avoid model overfitting. We optimized the negative binomial error distribution by fitting the most appropriate dispersion parameter (*theta*). We chose optimal *theta* for the final model by ranking models differing in *theta* (from 0.01 to 1.00). The model with the lowest AIC was assumed to be the best parametrized (on optimal *theta*)^60^. As the wind speed variable had an outlier value (6.9 m/s), we extracted the outlier and refitted GAM1 to check its influence on the results of GAM1. We found negligible effects of the tested outlier on model results, thus we decided not to remove it from the analyses.

We analyzed the effect of insect swarming (more than 10 insects in at least one photo out of 5 taken within 5 min) on bats feeding activity (i.e., feeding buzzes) by fitting a binomial generalized additive model (GAM2). We calculated the number feeding buzzes within 5 min. time window (range from 0 to 8). As most of the 5 min. periods lacked insects and feeding buzzes, we transformed these two variables into binomial variables insects: 1—presence, 0—absence; feeding buzzes: 1—presence, 0—absence). We set the presence of bat feeding buzzes as a binomial response variable and insect swarm presence as the explanatory variable. We used the same smoothing settings as in GAM1. We added meadow ID as a random effect (as a penalized regression term)^60^.

We validated model assumptions by visually inspecting validation plots of simulated model residuals provided by the “DHARMa” package^61^. Both qq-plots (detecting overall deviation from the expected distribution) and plots presenting residuals against predicted values confirmed that both models (GAM1 and GAM2) were specified correctly.

### Supplementary Information

Supplementary Information.

## Data Availability

Data files associated with this study are publicly available in the Open Research Data Repository of the Max Planck Society “Edmond” 10.17617/3.2PNZX2.
