# Supplementary material for: High temporal resolution data reveal low bat and insect activity over managed meadows in central Europe
Source: Sci Rep. 2024 Mar 29;14:7498. doi: 10.1038/s41598-024-57915-0 (PMC10980742; doi:10.1038/s41598-024-57915-0)
Supplement: Supplementary file 1 — Supplementary Information. [file 41598_2024_57915_MOESM1_ESM.pdf]

Supplementary Information

Supplementary Table S1: Ten different meadows ranked by the number of insects with the first meadow having the highest insect numbers (recorded per minute). Provided are the coordinates, size, distance to closest waterbody (m), distance to closest meadow, adjacent habitat, year of data collection, management of the meadow, max insect numbers, mean insect numbers and the standard deviation of insects per photo for each meadow, summarized for all nights and if data on bat buzzes were collected.

**Meadow**	**Coordinates**	**Size**	**Distance to closest waterbody**	**Distance to closest meadow**	**Adjacent habitat**	**2020**	**2021**	**Management**	**Insects.max**	**Insects.mean**	**Insects.sd**	**Buzzes**
1 Mill	47°45'51.5"N 8°59'55.3"E	33507 m^2	120.5 m to a brook	227 m	forest, wet grassland, small water body nearby	no	yes	hay meadow, fertilized	105	1,4	7,4	yes
2 Uni	47°41'21.6"N 9°11'02.5"E	3237 m^2	278.7 m to a brook	401 m	forest, houses, road	yes	yes	hay meadow	96	1,7	6,1	yes
3 Tannenhof	47°40'49.4"N 9°11'42.0"E	7654 m^2	107.1 m to a brook	820 m	settlement, rows of trees, shrubs, small water body nearby	yes	yes	hay meadow, football field nearby	75	1	3,9	yes
4 Hockgraben	47°41'12.8"N 9°11'18.2"E	8473 m^2	96.9 m to a pond	401 m	forest, small water body nearby	yes	yes	hay meadow	41	0,7	2,4	yes
5 StKatharina	47°41'47.6"N 9°10'25.9"E	7552 m^2	48.2 m to a brook	1140 m	forest clearing	yes	yes	hay meadow	37	1,2	3,1	yes

6 Scheune	47°45'56.3"N 8°59'56.5"E	14850 m^2	94.1 m to a pond	227 m	fruit trees, houses, road	yes	no	tilled grain field (soil)	37	1,1	2,7	yes
7 Voliere	47°45'53.4"N 8°59'44.3"E	1100 m^2	50.0 m to a brook	334 m	forest, houses	yes	yes	hay meadow, protected	36	0,8	1,9	yes
8 Institute	47°46'04.4"N 8°59'50.7"E	13284 m^2	106.6 to a pond	304 m	fruit trees, forest, road	yes	no	grazed by sheep 2 x year	15	0,8	1,5	yes
9 Hegne	47°42'26.6"N 9°05'43.3"E	25200 m^2	227.5 m to Lake Constance	3870 m	bushes, country road, lake nearby	yes	no	hay meadow, fertilized	13	0,4	0,9	no
10 Wollmatingen	47°41'15.8"N 9°08'30.5"E	6142 m^2	36.1 m to a brook	3250 m	houses, forest	yes	no	hay meadow, fertilized	7	0,3	0,8	no

Supplementary Table S2: Meadows ranked by the mean number of buzzes recorded per night of monitoring and mean number of buzzes per species and night. Note that we had only from 8 out of the 10 meadows acoustic data for feeding buzz analysis.

	n nights	mean buzzes per night	mean per species per night			
			Nyctalus noctula	*Pip nathusii/kuhlii*	*P. pipistrellus*	*P. pygmaeus*
StKatharina	10	15.5	0.5	0.4	7.2	7.2
Hockgraben	14	7.6	2.79	0.29	3.36	1.29
Mill	5	5,2	0	0.4	4	0.8
Voliere	5	4	0	0	3.4	0.6
Institut	4	2.3	0.25	0	2	0
Tannenhof	6	1.7	0.17	0	0.83	0.67
Uni	4	1.5	0	0	1	0.5
Scheunenwiese	5	1.4	0.2	0.4	0.6	0.2

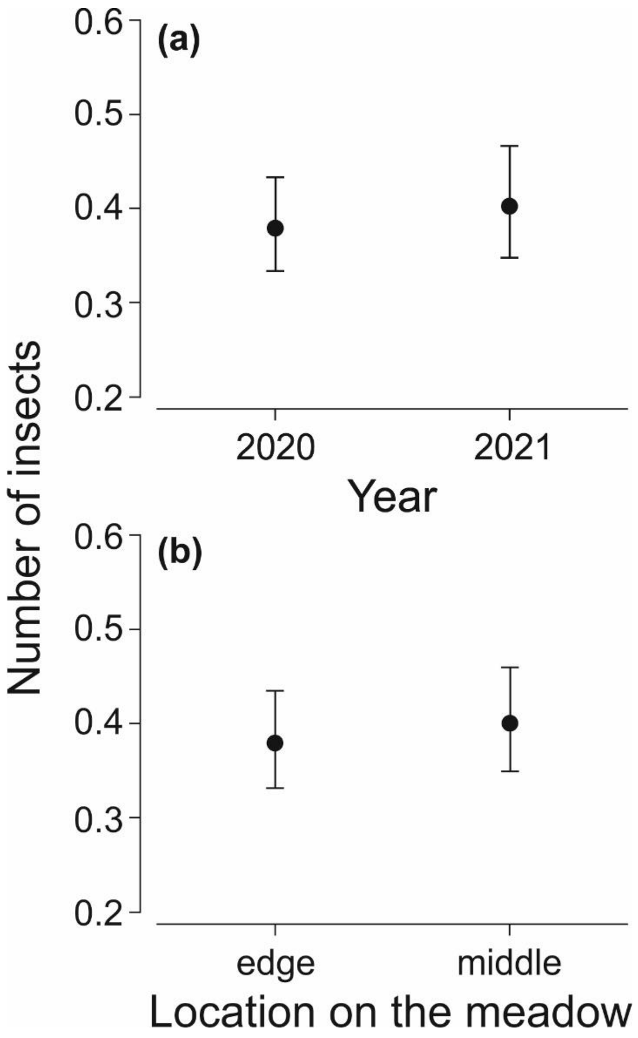

Supplementary Figure S1: Effect of year (a) and position on the meadow (b) on insect numbers. a) There was no difference in insect numbers between 2020 and 2021. b) A small difference in insect numbers between middle and edge of meadows was found. (Model output Generalized additive model; GAM1, Table 1).
